# Hymenoptera sting reactions in southern Italy forestry workers: our experience compared to reported data

**DOI:** 10.1186/s12948-018-0087-6

**Published:** 2018-04-17

**Authors:** Luisa Ricciardi, Francesco Papia, Giuseppe Cataldo, Mario Giorgianni, Giovanna Spatari, Sebastiano Gangemi

**Affiliations:** 1 Department of Clinical and Experimental Medicine, School and Division of Allergy and Clinical Immunology, University of Messina, Messina, Italy; 2 Department of Biomedical Sciences, Dental, Morphological and Functional Investigations, University of Messina, Messina, Italy

## Abstract

**Background:**

Hymenoptera sting reactions are among life-threatening causes of allergy. Several epidemiology studies have assessed the risk of these kind of reactions, among the general population, around 3% of adults. This incidence increases among highly at risk populations such as outdoor workers. Hymenoptera stings among forestry workers (FW) are occupational triggers but it has not yet been well defined which is the real incidence of anaphylaxis in these workers, not even in Italy. Two Italian studies reported on the risk of hymenoptera stings (HS) in northern Italy (NI) and central Italy (CI) FW while no data is available on the prevalence in southern Italy (SI) ones.

**Methods:**

A population of 341 SI FW (301 males and 40 females, mean age 51 years, range 43–63 years), who worked in Sicily, was investigated submitting a standardized questionnaire dealing with reactions to Hymenoptera stings, such as large local reactions (LLR) and systemic reactions (SR).

**Results:**

HS occurred in 203 FW (59%) and caused reactions in 77 (22%); LLR occurred in 46 (13%) and SR in 31 (9%); SR were life threatening in 9/341 (3%) FW and were treated with epinephrine at the emergency unit as workers did not carry an epinephrine auto-injector. A SR at a subsequent HS followed a LLR in 21/46 FW (46%).

**Conclusions:**

FW in SI have a generic risk of HS anaphylaxis as in the general population but a higher risk of SR and LLR respect to forestry populations from different Italian geographical areas.SR among SI FW occurred in 9% of them, while published data report the incidence of SR around 2 and 4%, respectively, in the Centre and North Italy FW. The incidence of LLR in SI FW was also higher (13%) than in CI (2%) and NI (10%) ones. Previous LLR in our SI population represented a high risk factor for developing a SR and therefore a red flag for future anaphylaxis and prescription of an epinephrine auto-injector.

## Background

Hymenoptera stings (HS), even if in the vast majority cause only minor problems, account, even nowadays in the third millennium, for deaths usually resulting from immunologic mechanisms.

Self-reported systemic HS reactions among adults range from 0.5 to 3.3% in the US [1] while in Europe studies report the prevalence of systemic reactions (SR) between 0.3 and 7.5% [2]; mortality due to HS has been reported ranging from 0.03 to 0.48 fatalities per 1,000,000 population per year [3].

Quality of life of subjects who have experienced a SR after a HS is impaired as these subjects usually develop emotional distress during day life [4]. Furthermore, HS are among the commonest triggers of occupational anaphylaxis especially in outdoor workers such as beekeepers [5], gardeners [6], farmers, truck drivers, masons [7] and forestry workers (FW) [8]. Some authors investigated the prevalence of reactions to HS among FW. Japanese FW have a percentage of SR to HS significantly higher than control subjects do [9]. Incorvaia [10] and Copertaro [11] studied northern Italy (NI) and central Italy (CI) populations of FW, respectively in order to evaluate the prevalence of HS. Up to now, no data is available on southern Italy (SI) FWs’ risk of HS reactions.

## Methods

We carried out an observational retrospective study on a population of FW from Sicily, a SI region, submitting a standardized fully anonymous questionnaire dealing with reactions to HS.

The reactions to HS were classified into large local reactions (LLR), defined as a swelling exceeding a diameter of 10 cm that lasted longer than 24 h, or SR according to Mueller’s classification [12] with skin, gastrointestinal, respiratory and cardiovascular systems’ involvement. Life threatening SR, defined as anaphylaxis, were the reactions characterized by a rapid onset of airway, breathing, circulatory, or gastrointestinal problems defined according to EAACI and WAO Guidelines [13, 14].

A physician administered a questionnaire to the FW in order to collect information about age, sex, HS, stinging insect, average number of stings respect to how long they had been working, frequency of stinging, degree of reaction to a HS.

## Results

The population of FW consisted of 341 workers, mean age 51 years (range 43–63 years), 301 males and 40 females; HS occurred in 203 FW (59%), all during working hours. The culprit Hymenoptera, recognized by each stung FW, was a Vespid in 108 and an Apid in nine workers. Stings received by the other FW were most likely from Vespids as they did nor remember to have removed the sting. In average FW included in the study had been working for 23 years. Since their employment, 64 workers had received from 1 to 3 stings, 86 between 3 and 5 while 53 more than 5. HS reactions occurred in 77 FW (22%). LLR occurred in 46 FW (13%) and about half of them, 21 (46%), after a second sting in a further occasion, had a SR. LLR were also more frequent in workers who medially had been working for more years. The overall number of FW who had a SR was of 31/341 (9%).

These reactions had been treated with topical or systemic corticosteroids or antihistamines. SR were life threatening in 9/341 (3%) and were treated with epinephrine at the emergency unit together with systemic antihistamines such as clorpheniramine and corticosteroids as methylprednisolone. Furthermore, all the workers who had life-threatening SR were among those who had received more than five stings. No FW carried an epinephrine auto-injector.

## Discussion

FW are at high risk of HS and may develop occupation-related allergies but rarely surveys on the natural history of HS, among these or other outdoor workers, are reported [15].

In Italy (Table 1) surveys on NI [10] and CI [11] FW, investigating the incidence of HS reactions in these populations, reported SR in 4 and 2% of FW, respectively while LLR occurred in 10 and 2%, respectively. Data were lacking on the incidence of HS reactions in SI FW and therefore we carried out the present survey.

**Table 1:** General data and kind of reaction to Hymenoptera stings in FW from SI (current report), CI and NI (as previously published 10, 11)

	FW	Mean age	Males	Females	Nº HS^a^	LLR	SR
FW SI	341	51	301	40	203 (59%)	46 (13%)	31 (9%)
FW CI	206	39.6	180	26	179 (87%)	4 (2%)	4 (2%)
FW NI	112	39	112	/	77 (68%)	11 (10%)	5 (4%)

^a^Hymenoptera stings

**Fig. 1:**
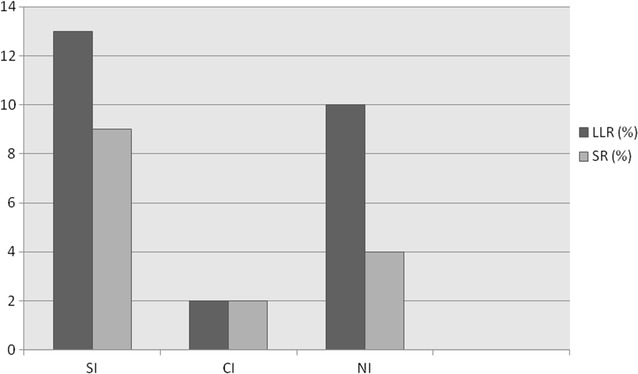
Percentage of LLR and SR in FW populations from SI, CI, NI

A higher incidence of SR (9%) and LLR (13%) in SI FW was shown, compared to NI and CI ones, even if a lower percentage of SI FW was stung (Fig. 1).

HS occurred in 203/341 SI FW (59%) compared to 76/112 (68%) in NI and 179/206 (87%) in CI ones. Nonetheless, among SI FW, only 64 workers received no more than three stings while 86 from 3 to 5 and 53 more than 5. The higher incidence of both systemic and LLR in SI FW could be correlated to the shortness of interval between stings [16]. Hymenoptera allergy is one of the allergic disease problems related to climate change which is involving also Sicily [17]. A warming climate can cause dramatic shifts on these insects’ populations from extinction but usually to overpopulation with a significant increase in the number of people seeking care for stings [18].

The high incidence of LLR in our FW population is unusually high compared to the general population as previously reported [19] but up to now we are unable to explain this singularity. Only in highly exposed subjects, such as beekeepers [20] or subjects from a rural population in the Mediterranean area [21] a prevalence has been reported. It must be underlined that Sicilian FW, such as the population we examined, work in a similar geographical area.

Our data confirm what already reported in literature on how strong impact, hymenoptera venom allergy, has on work causing in some cases work disability [22].

As far as LLR in SI FW, they not also occurred with a higher incidence (13%) but a SR followed at a subsequent HS (21/46) in a high percentage of FW (46%).

LLR in SI FW represented a high risk factor for developing a SR and consequently a red flag for future anaphylaxis with the need of an epinephrine auto-injector prescription beforehand [23]. This accounts for the suggestion of a thorough allergy screening and follow-up in subjects with a high occupational risk of Hymenoptera stinging.
